# The Louisville Review

**Published:** 1856-09

**Authors:** 


*The Louisville Review.*—This journal, which started with such favorable auspices a few months since, is to be removed to Philadelphia. Both the editors have recently been called to professorships in the Quaker city, and we are gratified to learn that the Review is not to pass from their able management. We presume some change of name will be found necessary, but we hope there will be none in the character of its conduct.

